# Metabonomics Study in Mice With Learning and Memory Impairment on the Intervention of Essential Oil Extracted From *Cinnamomum camphora* Chvar. Borneol

**DOI:** 10.3389/fphar.2022.770411

**Published:** 2022-03-10

**Authors:** Yin Tang, Xiaofan Lv, Yumin Liu, Donghong Cui, Yani Wu

**Affiliations:** ^1^ School of Design, Shanghai Jiao Tong University, Shanghai, China; ^2^ Analysis and Testing Centre of Shanghai Jiao Tong University, Shanghai, China; ^3^ Shanghai Key Laboratory of Psychiatric Disorders, Shanghai Mental Health Center, Shanghai Jiao Tong University School of Medicine, Shanghai, China

**Keywords:** essential oil of *Cinnamomum camphora* chvar. Borneol, learning and memory, metabonomics, differential metabolites, metabolic pathway

## Abstract

Our objective was to explore the mechanism of essential oil that was extracted from *Cinnamomum camphora* chvar. Borneol (Borneol essential oil) for improving learning and memory impairment in mice. Brain tissue and plasma samples of a normal group, a model group, a Borneol essential oil group and a reference group were detected using gas chromatography time-of-flight mass spectrometry (GC-TOFMS) in order to find differential metabolites and analyze metabolic pathways. Results showed that there were 11 different metabolites --including glycine and azelaic acid --in plasma samples, and that there were 26 different metabolites--including adenine and aspartic acid --in brain tissue samples. These metabolites are involved in phenylalanine, tyrosine, and tryptophan biosynthesis, phenylalanine metabolism, alanine, aspartate and glutamate metabolism, arginine biosynthesis, beta-alanine metabolism, glyoxylate acid and dicarboxylate metabolism, and aminoacyl-tRNA biosynthesis. Thus, Borneol essential oil may improve learning and memory impairment by regulating amino acid metabolism and/or neurotransmitter changes.

## Introduction

Metabonomics is one of the important research methods in systems biology. By analyzing a large number of endogenous metabolites in complex biological matrices, we can examine the mechanism of biochemical disturbances caused by diseases, drugs, or toxins (Phua et al., 2013). At present, metabolomics research can be completed using a variety of biological samples, including blood, feces, saliva, tissue, and urine. Gas chromatography time-of-flight mass spectrometry (GC-TOFMS) is a mature technology with fast ion collection speed and wide mass range that is suitable for the full spectrum of metabolomics analysis of biological substances (Liu et al., 2011).

Learning and memory impairment refers to the decline in the ability to remember, maintain, re-recognize and reproduce objective things (Gupta et al., 2018). A classic learning and memory disease, Alzheimer’s disease, has received increasing social attention in recent years. According to research reports in 2020, there were approximately 9.83 million Alzheimer’s disease patients in China (Jia et al., 2020). Thus, preventing and treating Alzheimer’s disease is urgent. At present, the clinical treatment of Alzheimer’s disease uses marketed drugs including donepezil and memantine. In addition, many scholars have found that rosemary essential oil (Liu et al., 2015; Filiptsova et al., 2017), citronella essential oil (Wang and Kang, 2017), and peppermint compound essential oil (Sun et al., 2019) can improve learning and memory impairment in rats and/or humans. Previous work by our team has also determined that, after sniffing *Cinnamomum camphora* chvar. Borneol essential oil (Borneol essential oil), mice’s scores on the Morris water maze, a classic index of learning and memory, improved, and that their hippocampal β-amyloid proteins decreased. Thus, it appears that Borneol essential oils can improve learning and memory impairment in mice. In this study, we collected brain tissue and plasma samples from mice, and used GC-TOFMS metabonomics to explore the physiological mechanism underlying Borneol essential oil’s effects on improving learning and memory impairment in mice.

## Materials

### Reagents and Equipment

Borneol essential oil and rosemary essential oil were provided by the aromatherapy laboratory of Shanghai Jiao Tong University. Methanol, chloroform, methoxyamine hydrochloride, pyridine, L-2-chlorophenylalanine, and n,o-bis-trimethylsilyl-trifluoroacetamide (BSTFA) that contained 1% trimethylchlorosilane (TMCS) were provided by the Analysis and Testing Centre of Shanghai Jiao Tong University.

Agilent 6890N gas chromatograph and Pegasus 4D TOF-MS were from the Laboratory Equipment Corporation (United States); the TGL-16M high-speed freezing centrifuge was from the Hunan Xiangyi Laboratory Instrument Company Limited (China), and the LNG-T88 vacuum centrifugal concentrator was from the Taicang Huamei Biochemical Instrument Factory (China).

### Animals and Groups

Six-month-old learning and memory impairment model mice induced by APP/PS1 transgenic (model mice) and normal C57BL/6J mice (normal mice) of the same genetic background were provided by the Changzhou Cavens Laboratory Animal Co., Ltd., with license number SCXK (Su) 2016-0010. We constructed a normal group which used normal mice. We constructed a model group, a Borneol essential oil group and a reference group, which used model mice. We had eight mice in each group. The mice in the Borneol essential oil group sniffed Borneol essential oil, and the mice in the reference group sniffed rosemary essential oil [efficacy was determined by animal and volunteer experiments (Liu et al., 2015; Filiptsova et al., 2017)], 2 times/day, 1 h each time, for 21 days. There was no treatment for the model group or the normal group. We uniformly collected metabonomics samples from all mice after 21 days.

## Methods

### Sample Collection and Pretreatment

#### Plasma Samples

The mice were anesthetized with ether, and blood was taken from their eyeballs. The blood was added to EDTA anticoagulant, centrifuged in the centrifuge for 15 min (4°C, 3,000 r/min), and the supernatant plasma was taken and stored in a −80°C ultra-low temperature refrigerator for later use. When in use, the plasma was thawed at room temperature. We added 100 μl plasma to centrifuge tube, and then added 300 μl methanol-chloroform (3:1, V/V) and 10 μl L-2-chlorophenylalanine (0.3 mg/ml). We then shook the centrifuge tube for 30 s, and placed it in a freezer at 20°C for 20 min. We next centrifuged the sample for 10 min (4°C, 12,000 r/min), taking 300 μl supernatant into a 1.5 ml autosampler vial, concentrated the sample in vacuum and dried it with nitrogen. We added 80 μl pyridine solution to methoxyamine hydrochloride (15 mg/ml), sealed the vial, and put the sample on a shaking bed for one night at room temperature. Then, we opened the vial, added 80 μl of BSTFA (including 1% TMCS), sealed the vial, and put the sample in a thermostatic box for 60 min at 70°C. We detected samples at room temperature (Liu et al., 2017).

#### Brain Tissue Samples

We decapitated the mice and removed their brain tissue. We washed the tissue with normal saline, and then added normal saline and ground it up to make a 10% volume brain tissue homogenate. After centrifuging the sample for 10 min (4°C, 3,500 rpm), we took out the supernatant and placed it in a −80°C ultra-low temperature refrigerator for later use. The following steps were basically the same as those outlined in *Plasma Samples*.

### Parameters of GC-TOFMS

The DB-5MS capillary column (length: 30 m, diameter: 250 μm, thickness: 0.25 μm) was used. We set the starting temperature of column to 80°C for 2 min, increased it to 180°C at 10°C/min, increased it to 240°C at 5°C/min, and then increased it to 290°C at 25°C/min for 9 min. The carrier gas was helium, and the flow rate was 1 ml/min. The interface and ion source temperatures were 270 and 220°C, respectively. The ionization mode of the mass spectrometer was set to EI, and the ionization voltage was 70 eV. The machine was scanned in a full scan mode, with a scanning range of 30–550 m/z. The plasma sample solvent was delayed by 7.5 min, and the brain tissue sample solvent was delayed by 7.6 min.

### Data Processing and Analysis

LECO chroma TOF software was used to process the original data, including peak area integration, baseline calibration, and baseline filtering, to obtain the mathematical matrix. We imported this matrix into the SIMCA software for multidimensional statistical analysis. Potential differential metabolites were screened according to variable importance in the projection (VIP) and t-tests. The specific standards were VIP > 1 and *p* < 0.05. The NIST database and databases from Shanghai Jiao Tong University analysis and the Testing Centre were used to identify substances. Finally, the MetaboAnalyst platform was used to investigate the involved metabolic pathways (Liu et al., 2017).

## Results

### Principal Component Analysis

**FIGURE 1:**
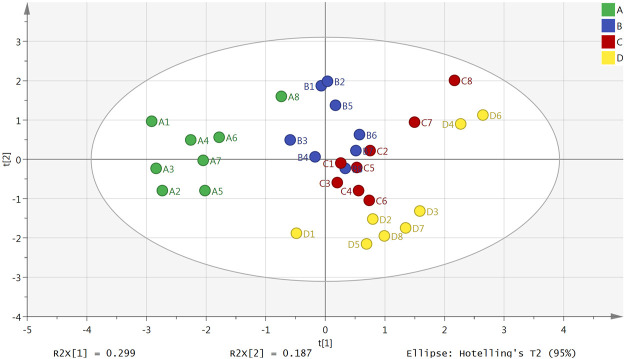
PCA scores plot of the normal group (A), model group (B), Borneol essential oil group (C), and reference group (D) in plasma samples (*n* = 8).

**FIGURE 2:**
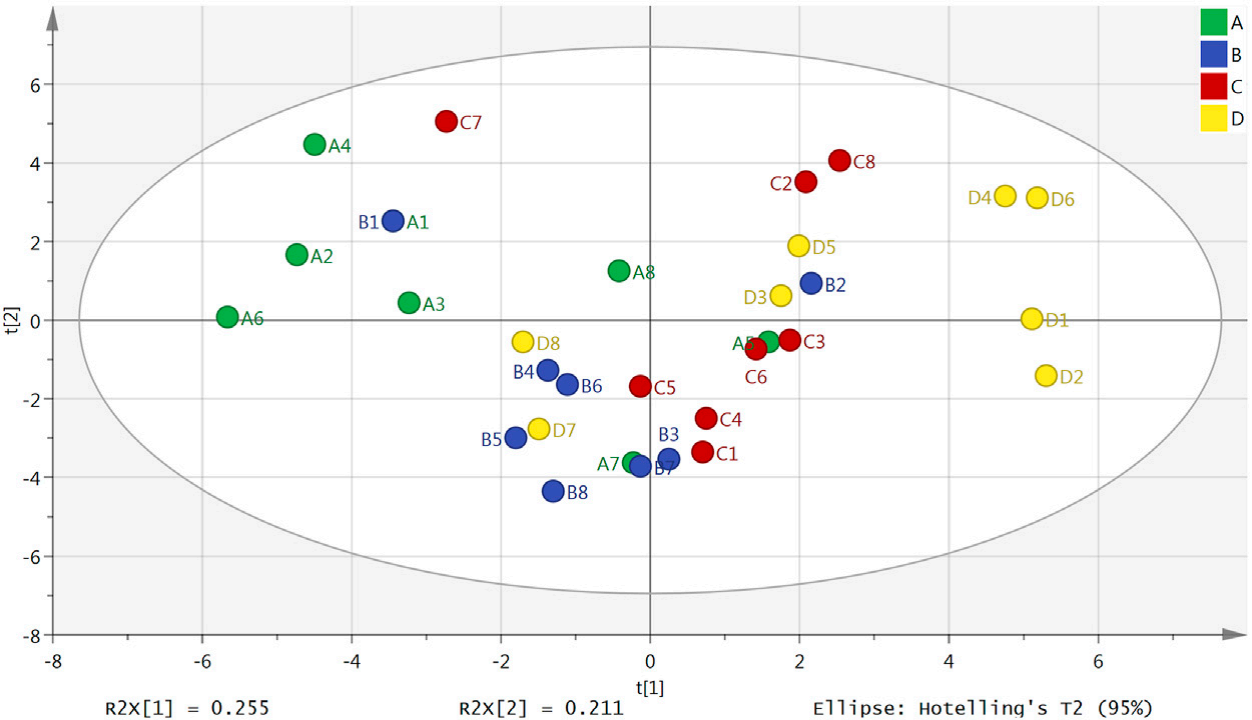
PCA scores plot of the normal group (A), model group (B), Borneol essential oil group (C), and reference group (D) in brain tissue samples (*n* = 8).

The result of a principal component analysis (PCA) showed that there were obvious differences between the plasma samples from the normal group, model group, Borneol essential oil group and the reference group (Figure 1). All samples were located within the 95% confidence interval, and the cumulative interpretation rate of the model was R^2^X = 0.687, Q^2^ = 0.134. There were also certain differences between the brain tissue samples in the four groups (Figure 2). All samples were located in 95% confidence interval, and the cumulative interpretation rate of the model was R^2^X = 0.665, Q^2^ = 0.392.

### Orthogonal Projections to Latent Structures Discriminant Analysis

**FIGURE 3:**
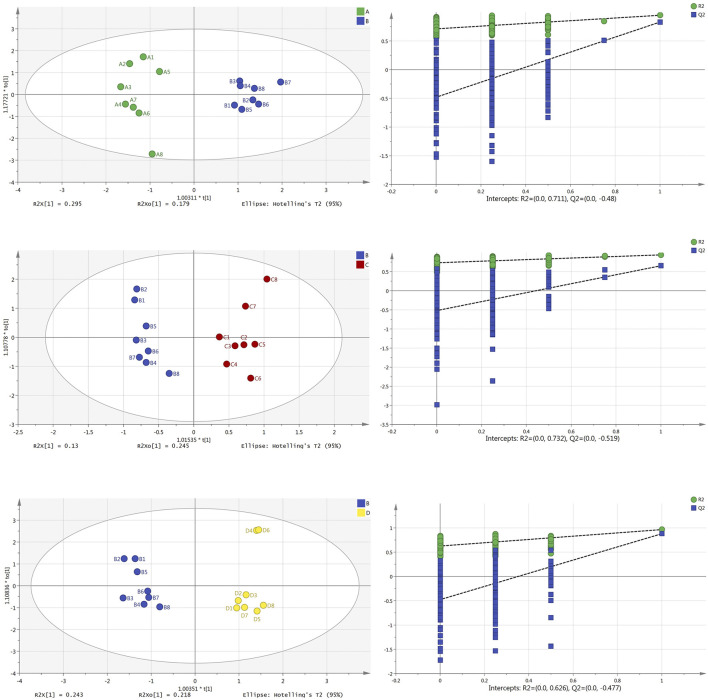
OPLS-DA scores plot and permutation test diagram of normal group (A), model group (B), Borneol essential oil group (C), and reference group (D) in plasma samples (*n* = 8).

In order to maximize the separation of samples and pay attention to the substances that contributed to model clustering, we used the supervised method of orthogonal projections to latent structures discriminant analysis (OPLS-DA) to establish the model again, and carried out permutation testing (*n* = 200) to evaluate whether the model was overfitted. The results from the plasma samples are shown in Figure 3: the OPLS-DA scores plot diagram of the normal group and model group (R^2^X = 0.474, R^2^Y = 0.948, Q^2^ = 0.830) and its permutation testing diagram (R^2^ = 0.711, Q^2^ = −0.480); the OPLS-DA scores plot diagram of the model group and Borneol essential oil group (R^2^X = 0.375, R^2^Y = 0.938, Q^2^ = 0.654) and its permutation testing diagram (R^2^ = 0.732, Q^2^ = −0.519); the OPLS-DA scores plot diagram of the model group and reference group (R^2^X = 0.461, R^2^Y = 0.964, Q^2^ = 0.879) and its permutation testing diagram (R^2^ = 0.626, Q^2^ = −0.477). Parameters indicate that these models are relatively stable, have good prediction abilities, and are not overfitted.

**FIGURE 4:**
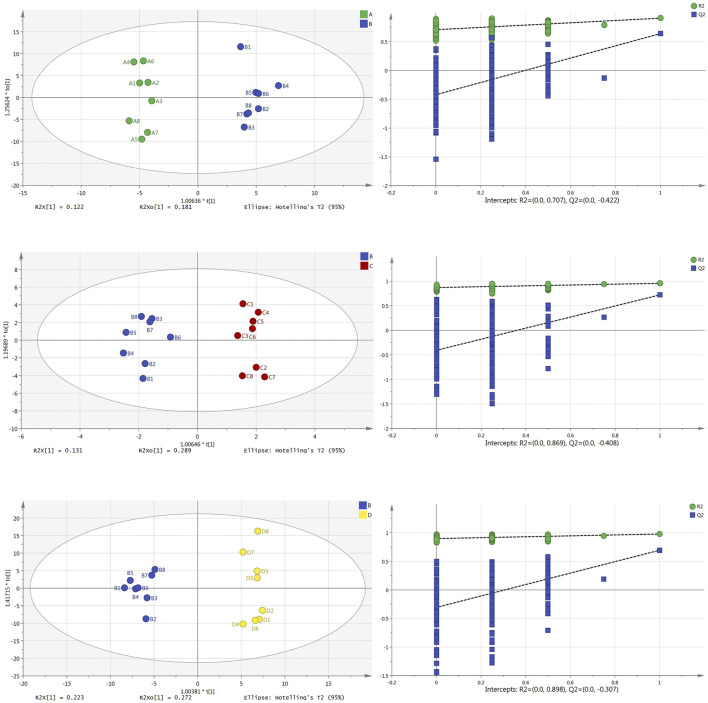
OPLS-DA scores plot and permutation test diagram of normal group (A), model group (B), Borneol essential oil group (C), and reference group (D) in brain tissue samples (*n* = 8).

Similarly, the parameter findings from brain tissue samples (Figure 4) show that all have good prediction abilities and are not overfitted. Main parameters: the OPLS-DA scores plot diagram of the normal group and model group (R^2^X = 0.421, R^2^Y = 0.973, Q^2^ = 0.483) and its permutation testing diagram (R^2^ = 0.707, Q^2^ = −0.422); the OPLS-DA scores plot diagram of the model group and Borneol essential oil group (R^2^X = 0.543, R^2^Y = 0.955, Q^2^ = 0.720) and its permutation testing diagram (R^2^ = 0.869, Q^2^ = −0.408); the OPLS-DA scores plot diagram of the model group and reference group (R^2^X = 0.513, R^2^Y = 0.907, Q^2^ = 0.639) and its permutation testing diagram (R^2^ = 0.898, Q^2^ = −0.307). Parameters indicate that those models are relatively stable, have good prediction abilities, and are not overfitted.

### Differential Metabolites

**FIGURE 5:**
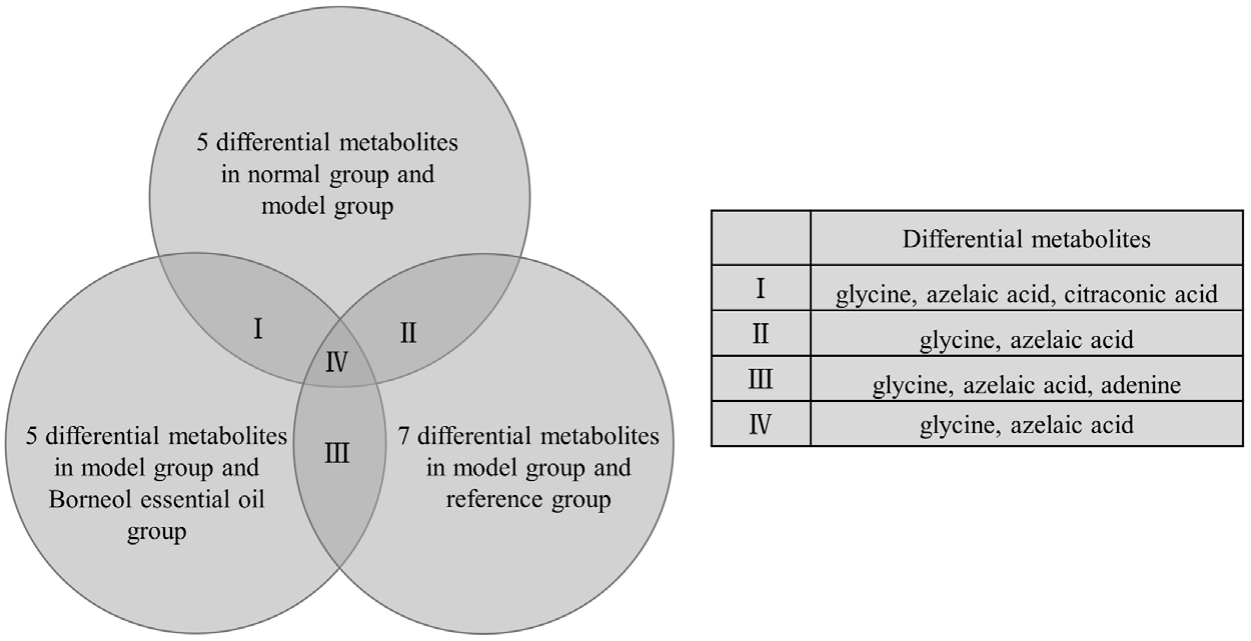
Differential metabolites repeated in plasma samples.

Under the above OPLS-DA model, according to VIP > 1 and *p* < 0.05, and referring to relevant research results to determine metabolites related to learning and memory. We find a total of 11 differential metabolites in plasma samples. There are five differential metabolites between the normal group and model group, five differential metabolites between the model group and Borneol essential oil group, and seven differential metabolites between the model group and the reference group. As shown in Figure 5, differential metabolites repeated amongst groups include glycine, azelaic acid, citraconic acid and adenine. Glycine and azelaic acid appear in all categories.

**FIGURE 6:**
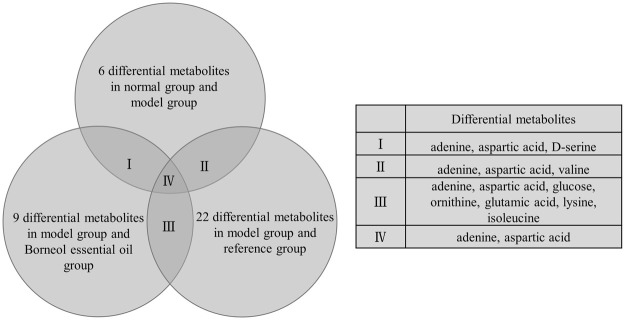
Differential metabolites repeated in brain tissue samples.

In brain tissue samples, 26 different metabolites in total were screened out. There were six differential metabolites between the normal group and the model group, nine differential metabolites between the model group and the Borneol essential oil group, and 22 differential metabolites between the model group and the reference group. From further analysis, we determined that differential metabolites repeated between multiple groups contained adenine, aspartic acid, D-serine, valine, glucose, ornithine, glutamic acid, lysine, and isoleucine. Adenine and aspartic acid appear in all categories, as shown in Figure 6. The specific data on differential Metabolites are shown in Supplementary Material.

### Metabolic Pathway

**FIGURE 7:**
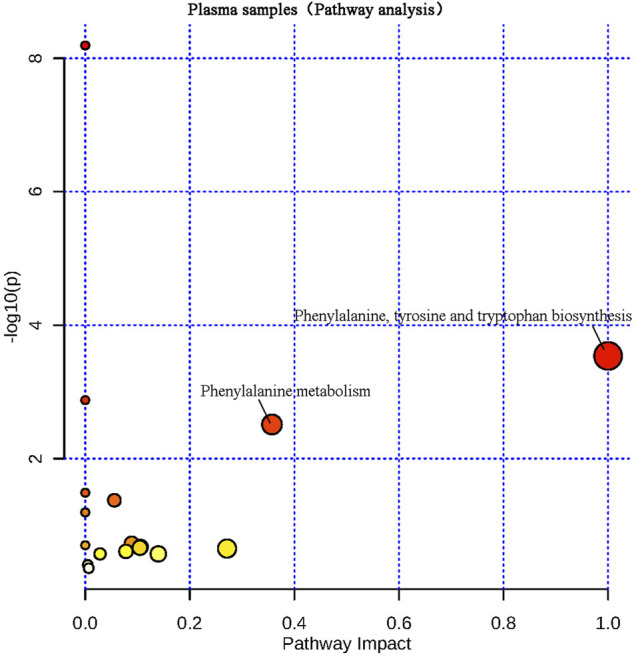
Metabolic pathway analysis of plasma samples.

**FIGURE 8:**
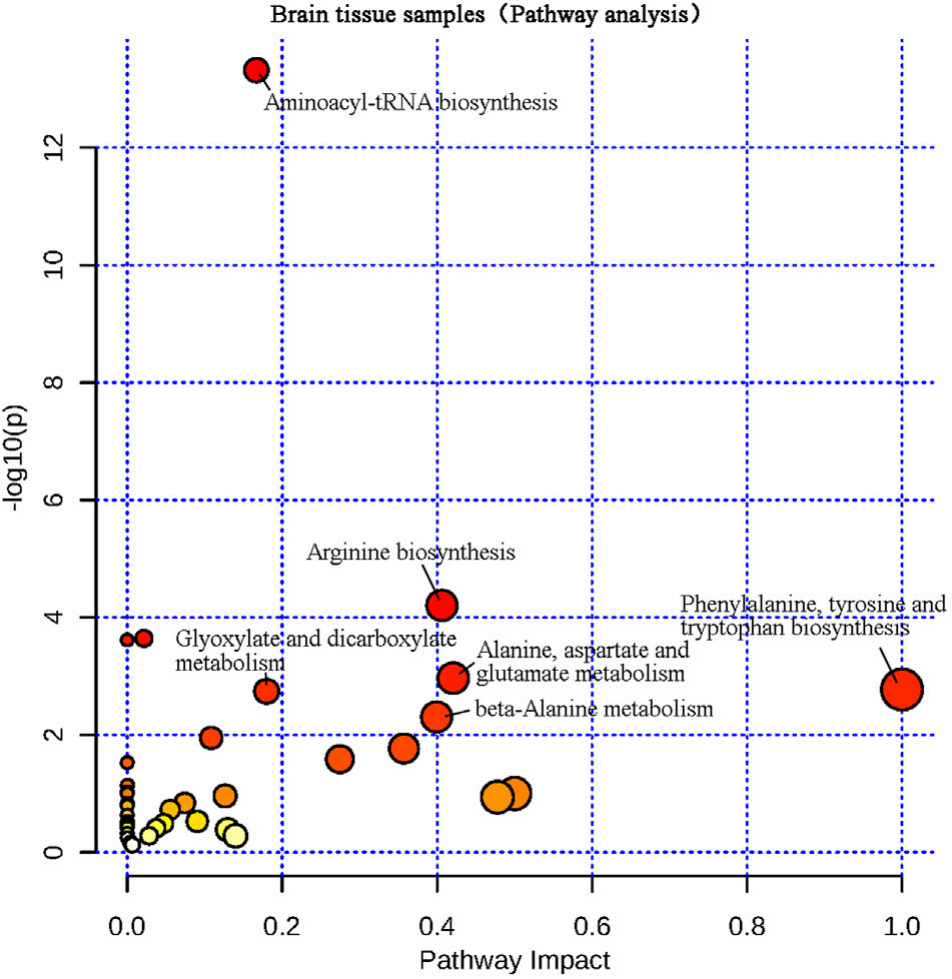
Metabolic pathway analysis of brain tissue samples.

The differential metabolites screened from plasma and brain tissue samples were put into the MetaboAnalyst 5.0 platform, and metabolic pathways related to learning and memory impairment were determined based on pathway impacts > 0.1 and *p* < 0.01 (−lg*P* > 2) (Liu et al., 2017). Results were shown in Figures 7, 8. Two metabolic pathways were found in plasma samples: phenylalanine, tyrosine and tryptophan biosynthesis, as well as phenylalanine metabolism. Six metabolic pathways are found in brain tissue samples: Phenylalanine, tyrosine and tryptophan biosynthesis; alanine, aspartate and glutamate metabolism; arginine biosynthesis; beta-alanine metabolism; glyoxylate and dicarboxylate metabolism; and aminoacyl-tRNA biosynthesis.

## Discussion

In plasma and brain tissue samples, five metabolic pathways--including phenylalanine, tyrosine and tryptophan biosynthesis, phenylalanine metabolism, alanine, aspartate and glutamate metabolism, arginine biosynthesis, and beta-alanine metabolism--belong to amino acid metabolism in KEGG pathway. As the constituent unit of protein, amino acids are closely related to various physiological activities (Wu, 2009). In this study, different metabolites of amino acids such as glycine, aspartic acid and D-serine were found. Previous studies have found that the content of amino acid neurotransmitters such as glycine and aspartate would fluctuate in models of learning and memory impairment (Wu and Zhang, 2018). Madeira et al. (2015) also found that the levels of D-serine and serine racemase (the enzyme responsible for producing D-serine) in the learning and memory impairment model rats were higher than those in the control group. D-serine can act on N-methyl-D-aspartate receptors, trigger signal transduction in the central nervous system, and also plays an important role in synaptic plasticity, learning and memory (Feng et al., 2013).

Glyoxylate and dicarboxylate metabolism is a part of carbohydrate metabolism in the KEGG pathway. Sun et al. comprehensively analyzed the endogenous metabolites in the urine of Alzheimer’s disease rats treated with Shengmai-San (a compound common in traditional Chinese Medicine), and found that glyoxylate and dicarboxylate metabolism was involved (Yi et al., 2017). Some scholars carried out a study on the effects of long-term iron exposure on cognitive functioning in C57/BL6 mice. They also found that glyoxylate and dicarboxylate metabolism was one of the differential metabolic pathways in the intestinal microbial spectrum of the control group and iron treatment group (Wang et al., 2019). In this study, the differential metabolites involved in glyoxylate and dicarboxylate metabolism are glutamic acid and citric acid. The synaptic transmission process involved in glutamic acid has an important impact on learning and memory (Esposito et al., 2013; Reiner and Levitz, 2018). Studies have found that glutamic acid function fluctuates during development from mild cognitive impairment to Alzheimer’s disease, and that glutamic acid content first decreases and then increases (Zhang and Ai, 2018). Citric acid is a metabolic intermediate of mitochondrial tricarboxylic acid cycle, and many scholars believe that mitochondrial dysfunction is one of the pathogeneses of Alzheimer’s disease (Perez-Ortiz and Swerdlow, 2019). We found that, compared with the normal group, citric acid content in the model group was decreased, which may be related to the abnormal mitochondrial functioning seen in learning and memory impairment model mice.

Aminoacyl-tRNA biosynthesis belongs to the genetic information processing component of the KEGG pathway. Some metabonomic studies on patients with Alzheimer’s disease have shown that aminoacyl-tRNA biosynthesis is an important involved metabolic pathway for the disease (Trushina et al., 2013; Wang et al., 2020). Aminoacyl-tRNA synthase and translation factors are key elements required for protein biosynthesis (Ibba and Söll, 2004). Mutation in aminoacyl-tRNA synthase is related to a variety of diseases, including cancer and various neurodegenerative diseases (i.e., Parkinson’s disease) (Sauter et al., 2015; Kapur et al., 2017). The exact relationship between learning and memory impairment and aminoacyl-tRNA synthesis is not clear, but studies have found that aminoacyl-tRNA synthase is related to the biosynthesis of dinucleotide phosphate (Zamecnik et al., 1966). Dinucleotide phosphate can act as a neurotransmitter to stimulate the release of γ-aminobutyric acid in the central and peripheral nervous systems, and is involved in responses to oxidative stress and metabolic changes, which are closely related to the onset of learning and memory impairment (Gómez-Villafuertes et al., 2004).

This study explored the effect of Borneol essential oil on plasma and brain tissue metabolites in mice with learning and memory impairment, which was similar to some study of natural products in the treatment of Alzheimer’s disease. Xia et al. (2017) studied the effect of breviscapine on Alzheimer disease mice by using HPLC-QTOF-MS metabolomics method, and found 10 potential biomarkers in plasmas, such as indoleacrylic acid, C16 sphinganine, and sulfolithocholic acid. From metabolic pathways analysis, they thought breviscapine improved the learning and memory deficits, which might be related to adjusting phospholipids metabolism. Gong et al. (2015) used UHPLC–TOF/MS method to determine the endogenous metabolites of plasma samples, finding the content change of proline, valine, tryptophan and so on in Alzheimer disease mice, and the levels of these metabolites were recovered in different degrees after total ginsenosides administration. At present, our study found differential metabolites and involved metabolic pathways, and further work is the verification and validation of markers. In recent years, muti-omics technology has been relatively mature, and has made progress in some studies. For example, a research integrated transcriptomic, proteomic and epigenomic analyses of postmortem humanbrains to identify molecular pathways involved in Alzheimer disease. And the identification of this process highlighted potential epigenetic strategies for early-stage disease treatment (Nativio et al., 2020). In the future, we can use the muti-omics technology to explore the internal mechanism of Borneol essential oil to improve learning and memory impairment in mice.

In conclusion, we found 11 differential metabolites--including glycine, azelaic acid, citraconic acid, adenine, methionine, isoleucine, cholesterol, tyrosine, proline, phenylalanine and leucine--in plasma samples, and 26 different metabolites --including adenine, aspartic acid, D-serine, valine, glucose, ornithine, glutamic acid, lysine, isoleucine, lactic acid, citric acid, gluconic acid, α-alanine, β-alanine, cholesterol, citrulline, glycine, leucine, methionine, myo-inositol, myristic acid, oleic acid, phenylalanine, proline, tyrosine and uracil in brain tissue samples. These differential metabolites are involved in phenylalanine, tyrosine and tryptophan biosynthesis, phenylalanine metabolism, alanine, aspartate and glutamate metabolism, arginine biosynthesis, and beta-alanine metabolism, glyoxylate and dicarboxylate metabolism and aminoacyl-tRNA biosynthesis. Therefore, in concert with relevant databases and research, we deduce that Borneol essential oil may improve the learning and memory impairment in mice by regulating amino acid metabolism and changes in neurotransmitter composition.

## Data Availability

The raw data supporting the conclusion of this article will be made available by the authors, without undue reservation.
