# Supplementary material for: Metabonomics Study in Mice With Learning and Memory Impairment on the Intervention of Essential Oil Extracted From Cinnamomum camphora Chvar. Borneol
Source: Front Pharmacol. 2022 Mar 10;13:770411. doi: 10.3389/fphar.2022.770411 (PMC8960444; doi:10.3389/fphar.2022.770411)
Supplement: Supplementary file 1 [file Table1.DOCX]

Supplementary Material

Differential metabolites of plasma samples (*n* = 8)

| Differential metabolites | normal group VS model group | | |  | model group VS Borneol essential oil group | | |  | model group VS reference group | | |
| --- | --- | --- | --- | --- | --- | --- | --- | --- | --- | --- | --- |
|  | VIP | *P* | Trend |  | VIP | *P* | Trend |  | VIP | *P* | Trend |
| Glycine | 1.30 | 1.99×10^-6^ | ↓ |  | 1.79 | 4.07×10^-5^ | ↓ |  | 1.01 | 5.82×10^-3^ | ↑ |
| Azelaic acid | 1.68 | 4.83×10^-3^ | ↓ |  | 1.56 | 3.12×10^-3^ | ↓ |  | 1.55 | 1.21×10^-4^ | ↓ |
| Citraconic acid | 2.04 | 4.78×10^-8^ | ↓ |  | 1.10 | 1.26×10^-3^ | ↓ |  |  |  |  |
| Adenine |  |  |  |  | 1.45 | 2.31×10^-8^ | ↑ |  | 1.68 | 7.35×10^-13^ | ↑ |
| Methionine | 1.24 | 1.79×10^-2^ | ↓ |  |  |  |  |  |  |  |  |
| Isoleucine | 1.01 | 2.53×10^-3^ | ↓ |  |  |  |  |  |  |  |  |
| Cholesterol |  |  |  |  | 2.12 | 4.91×10^-2^ | ↑ |  |  |  |  |
| Tyrosine |  |  |  |  |  |  |  |  | 1.27 | 1.50×10^-2^ | ↑ |
| Proline |  |  |  |  |  |  |  |  | 1.07 | 2.37×10^-2^ | ↑ |
| Phenylalanine |  |  |  |  |  |  |  |  | 1.03 | 1.69×10^-2^ | ↑ |
| Leucine |  |  |  |  |  |  |  |  | 1.01 | 2.37×10^-2^ | ↑ |

Differential metabolites of brain tissue samples (*n* = 8)

| Differential metabolites | normal group VS model group | | |  | model group VS Borneol essential oil group | | |  | model group VS reference group | | |
| --- | --- | --- | --- | --- | --- | --- | --- | --- | --- | --- | --- |
|  | VIP | *P* | Trend |  | VIP | *P* | Trend |  | VIP | *P* | Trend |
| Adenine | 2.63 | 2.34×10^-2^ | ↓ |  | 1.95 | 5.67×10^-3^ | ↓ |  | 1.36 | 5.18×10^-3^ | ↓ |
| Aspartic acid | 2.08 | 1.53×10^-2^ | ↑ |  | 1.91 | 4.85×10^-2^ | ↓ |  | 1.21 | 1.19×10^-2^ | ↑ |
| D-Serine | 1.18 | 4.39×10^-2^ | ↑ |  | 2.05 | 1.77×10^-2^ | ↓ |  |  |  |  |
| Valine | 1.98 | 1.53×10^-2^ | ↓ |  |  |  |  |  | 1.32 | 3.00×10^-2^ | ↓ |
| Glucose |  |  |  |  | 1.77 | 3.24×10^-2^ | ↓ |  | 1.02 | 3.15×10^-2^ | ↓ |
| Ornithine |  |  |  |  | 1.59 | 6.17×10^-3^ | ↓ |  | 1.36 | 9.30×10^-3^ | ↓ |
| Glutamic acid |  |  |  |  | 1.55 | 2.09×10^-3^ | ↓ |  | 1.43 | 2.50×10^-3^ | ↓ |
| Lysine |  |  |  |  | 1.15 | 1.28×10^-3^ | ↓ |  | 1.12 | 3.83×10^-2^ | ↓ |
| Isoleucine |  |  |  |  | 1.02 | 3.20×10^-4^ | ↑ |  | 1.49 | 2.31×10^-3^ | ↓ |
| Lactic acid | 2.40 | 2.42×10^-4^ | ↓ |  |  |  |  |  |  |  |  |
| Citric acid | 1.21 | 1.31×10^-2^ | ↓ |  |  |  |  |  |  |  |  |
| Gluconic acid |  |  |  |  | 2.16 | 4.72×10^-2^ | ↓ |  |  |  |  |
| α-Alanine |  |  |  |  |  |  |  |  | 1.10 | 2.64×10^-2^ | ↓ |
| β-Alanine |  |  |  |  |  |  |  |  | 1.24 | 1.72×10^-2^ | ↑ |
| Cholesterol |  |  |  |  |  |  |  |  | 1.58 | 1.63×10^-3^ | ↓ |
| Citrulline |  |  |  |  |  |  |  |  | 1.48 | 3.23×10^-3^ | ↓ |
| Glycine |  |  |  |  |  |  |  |  | 1.46 | 1.90×10^-3^ | ↓ |
| Leucine |  |  |  |  |  |  |  |  | 1.51 | 2.25×10^-3^ | ↓ |
| Methionine |  |  |  |  |  |  |  |  | 1.53 | 1.92×10^-3^ | ↓ |
| Myo-Inositol |  |  |  |  |  |  |  |  | 1.26 | 1.29×10^-2^ | ↓ |
| Myristic acid |  |  |  |  |  |  |  |  | 1.58 | 3.75×10^-3^ | ↓ |
| Oleic acid |  |  |  |  |  |  |  |  | 1.32 | 2.92×10^-2^ | ↓ |
| Phenylalanine |  |  |  |  |  |  |  |  | 1.57 | 1.37×10^-3^ | ↓ |
| Proline |  |  |  |  |  |  |  |  | 1.42 | 4.39×10^-3^ | ↓ |
| Tyrosine |  |  |  |  |  |  |  |  | 1.49 | 2.95×10^-3^ | ↓ |
| Uracil |  |  |  |  |  |  |  |  | 1.02 | 3.07×10^-2^ | ↓ |
